# Human Health Risk Assessment of Pharmaceuticals in Water: Issues and Challenges Ahead

**DOI:** 10.3390/ijerph7113929

**Published:** 2010-11-05

**Authors:** Arun Kumar, Biao Chang, Irene Xagoraraki

**Affiliations:** 1 Department of Civil Engineering, Indian Institute of Technology Delhi, Hauz Khas, New Delhi 110016, India; E-Mail: arunku@civil.iitd.ac.in; 2 School of Civil and Environmental Engineering, Georgia Institute of Technology, 215 Sustainable Education Building, 788 Atlantic Drive NW, Atlanta, GA 30332, USA; E-Mail: bchang6@gatech.edu; 3 Department of Civil and Environmental Engineering, A124 Engineering Research Complex, Michigan State University, East Lansing, MI 48824, USA

**Keywords:** human health, pharmaceuticals, mixture toxicity, risk assessment, uncertainty

## Abstract

This study identified existing issues related to quantitative pharmaceutical risk assessment (QPhRA, hereafter) for pharmaceuticals in water and proposed possible solutions by analyzing methodologies and findings of different published QPhRA studies. Retrospective site-specific QPhRA studies from different parts of the world (U.S.A., United Kingdom, Europe, India, *etc.*) were reviewed in a structured manner to understand different assumptions, outcomes obtained and issues, identified/addressed/raised by the different QPhRA studies. Till date, most of the published studies have concluded that there is no appreciable risk to human health during environmental exposures of pharmaceuticals; however, attention is still required to following identified issues: (1) Use of measured *versus* predicted pharmaceutical concentration, (2) Identification of pharmaceuticals-of-concern and compounds needing special considerations, (3) Use of source water *versus* finished drinking water-related exposure scenarios, (4) Selection of representative exposure routes, (5) Valuation of uncertainty factors, and (6) Risk assessment for mixture of chemicals. To close the existing data and methodology gaps, this study proposed possible ways to address and/or incorporation these considerations within the QPhRA framework; however, more research work is still required to address issues, such as incorporation of short-term to long-term extrapolation and mixture effects in the QPhRA framework. Specifically, this study proposed a development of a new “mixture effects-related uncertainty factor” for mixture of chemicals (*i.e.*, mixUF_composite_), similar to an uncertainty factor of a single chemical, within the QPhRA framework. In addition to all five traditionally used uncertainty factors, this uncertainty factor is also proposed to include concentration effects due to presence of different range of concentration levels of pharmaceuticals in a mixture. However, further work is required to determine values of all six uncertainty factors and incorporate them to use during estimation of point-of-departure values within the QPhRA framework.

## 1. Introduction

**Figure 1:**
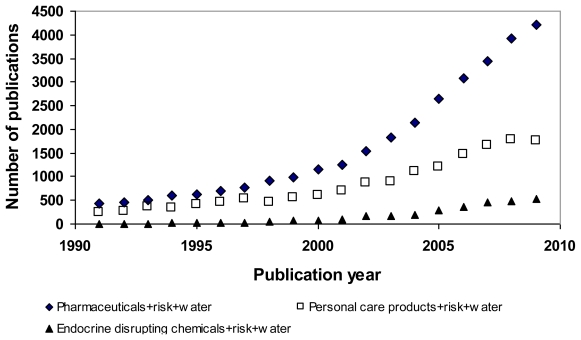
Search results using keywords shown in legends from the “ScienceDirect” database on December 31, 2009. Note: For “Pharmaceuticals + risk + water” keyword, total of 39,039 articles were found. For “Personal care products+risk+water” keyword, total of 20,438 articles were found. For “Endocrine disrupting chemicals + risk + water”, total of 3,601 articles were found.

In recent years, pharmaceuticals in water have received growing attention from environmental and health agencies all over the world and have become one of the emerging pollutants due to their frequent detection in the water environment [1–5]. The fact that pharmaceuticals are manufactured with the intention to cause biological effects has raised concerns about the impacts of unintentional pharmaceutical exposure on the health of human and ecological communities. Despite the relatively fast growing numbers of studies on ecological/environmental risk associated with pharmaceuticals in water, the number of publications related to studies on human health risks remains small (Figure 1), however, the trend is increasing with time. Even though risk from exposure to pharmaceuticals in drinking water is minimal [3,6–12], information about characterization of pharmaceuticals is still lacking.

In addition because of increasing public concern regarding potential health effects due to presence of pharmaceuticals in environment [13–15], it becomes important to understand and analyze different aspects of pharmaceutical exposures to humans, the associated health risks, and existing knowledge and data gaps.

The objective of this study is to identify existing issues within the quantitative pharmaceutical risk assessment (QPhRA, hereafter) framework by analyzing published risk assessment methodologies and frameworks and propose possible suggestions and research needs. Findings of this study are expected to highlight existing issues within the QPhRA framework and help in shaping future research directions towards filling the data and methodology gaps.

## 2. Identification of Existing Issues

This study (1) reviewed retrospective site-specific quantitative pharmaceuticals risk assessment (QPhRA) studies from different parts of the world (U.S.A., United Kingdom, Europe, India, *etc.*), (2) Analyzed information about four steps of the QPhRA methodology, and (3) Analyzed different identified/addressed/raised issues by different studies. The QPhRA process helps in estimating the nature and probability of adverse health effects in humans who may be exposed to pharmaceuticals from contaminated environmental media [16]. It primarily involves four major steps: (1) Hazard identification, (2) Exposure assessment, (3) Dose-response relationship, and (4) Risk characterization [3,6–12,15–18]. The reviewed retrospective site-specific QPhRA are summarized in Table 1. Following sections briefly discuss the stages of the QPhRA and related existing issues, needing more attention. It is important to note here that this list presents a brief summary of QPhRA studies highlighting different QPhRA steps and methodologies used by these studies and it does not necessarily represent the complete list of all QPhRA studies published so far.

### 2.1. Hazard Identification

#### 2.1.1. Measured *versus* predicted pharmaceutical concentration

Generally, both detected and modeled pharmaceutical concentrations are used in estimating risk for humans due to pharmaceuticals in water. The different pharmaceuticals studied can be seen in Table 1. With the advancement of detection techniques for pharmaceuticals in the environment, there are extensive published data on the occurrence of pharmaceuticals in water [4,18], some of which have been used in risk assessment studies. For example, the results of the national reconnaissance for pharmaceuticals and other contaminants in U.S. streams conducted by the U.S. Geological Survey during 1999 and 2000 [23] was used by Schwab *et al.* [3] to conduct the risk assessment. Also, Benotti *et al.* [1] analyzed 20 pharmaceuticals in source water, finished drinking water, and distribution system water from 19 U.S. water utilities between 2006 and 2007. These occurrence data are useful for conducting QPhRA, especially for the assessment of risks associated with drinking water consumption.

Modeled pharmaceuticals concentrations in water have also been used in different QPhRA studies. For example, two commonly used models are: (1) Pharmaceutical Assessment and Transport Evaluation (PhATE) [24] and (2) Geography-referenced Regional Exposure Assessment Tool for European Rivers [GREAT-ER,17,25]. The PhATE model is used to obtain predicted environmental concentrations (PECs) of active pharmaceutical ingredients (APIs) that results from patient’s use of medicines in 11 watersheds selected to be representative of most hydrologic regions of the U.S. [24]. It estimates values of PECs at drinking water locations and at stream segments based on flow summary statistics. The GREAT-ER model estimates concentrations of pharmaceuticals in stream segments of ten watersheds in Belgium, France, Germany, the U.K. and Netherlands by using Monte Carlo simulation to generate distributions of concentrations in segments which reflect the variability of various model parameters [8,17].

In general, modeled pharmaceutical concentrations are used in the case of no availability of appropriate concentration data and are useful for those substances which might be present at very low levels in environment. However, very few studies have validated these models before using for risk assessment purposes, suggesting the need for extensive model validation with field observed pharmaceutical concentration data. Further, proper considerations are also important for pharmaceuticals with low environmental concentrations due to problems with analytical detection methods.

#### 2.1.2. Pharmaceuticals-of-concern

The review of pharmaceuticals studied during different QPhRA studies, summarized in Table 1, indicates that studies have conducted risk assessment on a diverse range of pharmaceuticals in environmental waters depending on multiple criteria, such as occurrence, analytical capability, chemical properties, public perception, and possible health effects [3,6–12,15–18]. The selection of important pharmaceuticals in water depending on multiple criteria and subsequent risk assessment is a complex task. Although, different prioritization approaches are available for identifying pharmaceuticals-of-concern in both stream water and finished drinking water [10,22], they have not yet been integrated with the QPhRA framework.

The integration of pharmaceutical prioritization frameworks with the QPhRA frameworks has the potential of providing a holistic tool to different stakeholders for conducting pharmaceuticals risk assessment on a priority-basis. With regards to types of pharmaceuticals, a constant update of the pharmaceuticals list is required to include all newly detected pharmaceuticals, such as different metabolites and transformed products from parent organic compounds [26–28], depending on advancement of analytical methods.

It is worth noting here that the list of emerging organic compounds detected in environmental waters is increasing everyday due to improved detection of metabolites and transformed products from parent compounds [28], and this, frequent update of prioritized list is required to reflect occurrence of newly detected compounds in environmental waters. The developed lists should be region-specific reflecting concentration profiles of pharmaceuticals on a water body-level. Findings of the Larsson *et al.* [14] study illustrated the need for taking this kind of prioritization approach, where they reported very high levels of pharmaceuticals in wastewater effluent in Pantanchery (INDIA), with the most abundant pharmaceutical ciprofloxacin reaching up to 31,000 μg/L concentration level. Further, Schwab *et al.* [3] also proposed to consider regional effect during prediction of pharmaceuticals consumption and thus excreted pharmaceutical concentration levels in domestic wastewater. These reported findings illustrate the need for conducting water body-level QPhRA and avoid the practice of generalization the QPhRA risk estimates for different exposure scenarios.

### 2.2. Exposure Assessment

#### 2.2.1. Exposure routes

Most of the previous QPhRA studies have used scenario evaluation-based approach for estimating risk using different assumptions for developing exposure [3,6–12,17–21] (Table 1). This approach is one of the three U.S. EPA recommended approaches for exposure assessment [29] and it involves consideration of exposures through oral ingestion route, which generally happens during consumption of finished drinking water and/or fish (Table 1). This approach is consistent with the criteria used for estimating predicted-no-effect-concentration (PNEC) for pharmaceuticals in water [30]; however, other indirect exposure scenarios are also possible. For example, exposure to pharmaceuticals could also occur in following scenarios: (1) Indirect ingestion of food crops and/or vegetables irrigated with reclaimed wastewater or grown on sewage-sludge-amended soil [2], (2) Inhalation of pharmaceuticals during application of reclaimed wastewater for irrigation purposes, and (3) Dermal exposure. Most of the previous QPhRA studies have generally ignored these exposure scenarios, assuming a relatively smaller risk estimate from these scenarios compared to direct exposure scenario [3,10], which could be valid for pharmaceuticals with low vapor pressure but not for different class of pharmaceuticals. Review of most of the QPhRA studies indicates that comprehensive risk assessment studies including different exposure routes are required for different classes of pharmaceuticals.

#### 2.2.2. Exposure-related parameters

Exposure-related parameters (such as human ingestion rate and exposure duration) have been generally obtained using scenario-specific information during the hazard identification step and also using information given in the U.S. EPA Exposure Factors Handbook [31]. In addition, assumptions are also made in the absence of exposure-related data for pharmaceutical concentration, exposure frequency, and exposure duration. For example, studies have used surface water pharmaceutical concentration for estimating risk due to pharmaceuticals in finished drinking water, assuming that the drinking water treatment plant (DWTP) does not remove any pharmaceuticals from surface water [3,8,11,12,32]. Although, this approach is a conservative way of checking if there is any risk due to surface water, it does not represent the effect of water treatment plant on fate of pharmaceuticals and possible production of any other metabolites, which might be more dangerous than the parent pharmaceutical compound [12,26]. For example, a brief review of removal of different pharmaceuticals from full-scale DWTPs (Table 2) shows that some of the pharmaceutical compounds are removed completely from water whereas some of other pharmaceutical compounds are persistent in water in a conventional DWTP. Also, most of the pharmaceutical compounds investigated were found to be removed more than 90% from water in advanced DWTP. These findings indicate that the effect of removal effectiveness of different plant types should be included in QPhRA. For the case of low pharmaceutical-based exposure risks from stream water, related pharmaceutical-based exposure risks from finished drinking water would also be smaller due to effect of DWTP in removing pharmaceuticals from water. Considering this aspect, use of a source-specific pharmaceutical concentration is recommended for risk estimation purposes.

### 2.3. Dose-Response Relationship

#### 2.3.1. Uncertainty factors

This step involves estimation of (1) response values (*i.e.*, chronic daily intake (CDI) of pharmaceutical compounds) using exposed pharmaceutical dose and (2) benchmark values to compare calculated exposed pharmaceutical dose. For non-cancer effects, the exposed dose is usually compared with a health-based limit (HBL) (such as reference dose (RfD) or acceptable daily intake (ADI)); for carcinogenic effects, the dose is usually compared with a risk-specific dose (RSD) (*i.e.*, dose associated with a target risk, for example 10^−6^, 10^−5^, *etc*.) and estimations of these benchmark values generally involve utilization of results from previous toxicity assessment studies [32] and characterization of safety/uncertainty factors (UFs).

Generally, uncertainty factors have been classified into five categories: (1) Intraspecies variability (*i.e.*, human to human) (UF_1_), (2) Interspecies variability (*i.e.*, extrapolation from animal to human) (UF_2_), (3) Extrapolation from subchronic to chronic exposure (UF_3_), (4) Extrapolation from low-observable-adverse-effect-level (LOAEL) estimate to no-observable-adverse-effect-level (NOAEL) estimate (UF_4_), (5) Database quality and extrapolation (UF_5_), and a modifying factor based on professional assessment (MF). A review of different QPhRA studies indicated that the degree to which UFs were investigated and utilized varied significantly. Unlike NOEL estimates determined from toxicity studies, selection of UF values involve more subjective judgment [3] and presents a state of difficulty. Some studies have used default values of different UFs, depending on types of uncertainties they represent. For example, Schulman *et al.* [11] considered only UF_1_ and UF_4_ types of uncertainties and used a default value of 30 for the combination of these two factors. Schwab *et al.* [3], Cunningham *et al.* [8], and Kumar and Xagoraraki [10] discussed all five types of UFs and incorporated them into the development of ADIs.

Recently, some researchers have advocated the derivation and use of non-default values for UFs [3,6]. For example, Bercu *et al.* [6] essentially used UF_1_, UF_2_ and UF_4_ types of uncertainty factors in their risk assessment studies and used non-default values for some of these UFs. The use of non-default values of UFs for deriving estimates of ADIs appears to be a more representative approach as it does not include any extrapolation-based assumption and it determines values of UFs depending on uncertainty type and other considerations. Depending on availability of pharmaceutical-based data, non-default values representing toxicodynamics and toxicokinetics of different pharmaceuticals (*i.e.*, chemical-specific adjustment factors) should be used [6,26].

In addition, the uncertainty of long-term/chronic effects associated with exposure to pharmaceuticals in water has also been mentioned in most of the previous QPhRA studies (Table 1). Considerations of interactions of exposure duration and environmental pharmaceuticals concentrations become important due to the fact that some pharmaceuticals are designed to achieve acute effects and some pharmaceuticals are designed to achieve chronic effects. Although, the current QPhRA methodology uses uncertainty factor (UF_3_) to account for sub-chronic to chronic exposure extrapolation long-term effects might occur at relatively lower concentrations than those tested in toxicity experiments and might follow different toxicodynamic mechanisms than those extrapolated from short-term studies [20]. Thus, more long-term toxicity studies or experimental-simulation based hybrid approach are required to predict long-term toxicity effects.

#### 2.3.2. Endpoints

A chemical may elicit more than one toxic effect (*i.e.*, endpoint), even in one test animal, resulting in different NOEL values corresponding to different effects [32]. Generally, the identification of toxicological properties of a given pharmaceutical during QPhRA may include analyses of all possible health endpoints. However, due to constraints of time and resources, an in-depth analysis is rarely carried out for each health endpoint. For certain pharmaceuticals, endpoints might be defined from different types of experiments to further calculate values of ADIs. Uncertainties exist with the choice of endpoint and thus with the estimation of ADI values [3]. For example, Webb *et al.* [12] used toxicologically-based ADI, microbiologically-based ADI, pharmacologically-based ADI, and also therapeutic dosage as estimate of ADI similarly to other studies [6,8,11].

A direct consequence of identification of different endpoints is the generation of different ADIs for chemicals, which can be a source of considerable confusion when the ADIs are used exclusively in risk management decision making [32]. The use of different approximations for calculating ADI estimates poses an uncertainty in risk estimates, needing proper consideration. Theoretically, the critical endpoint used in the dose-response assessment should be the effect exhibiting the lowest NOEL [32]. However, in the previous practice of QPhRA, significant differences often exist between the ADIs of the same pharmaceutical calculated by different studies (Table 3). For example, for antibiotics such as doxycycline, tetracycline, and oxytetracycline, Webb *et al.* [12] used 3μg/kg/d whereas Schwab *et al.* [3] used 30 μg/kg/d as estimates of ADI values for each of these three antibiotics (Table 3). The primary reason of discrepancy of ADI estimates between these two studies was that Webb *et al.* [12] used therapeutic doses for estimating ADI values whereas Schwab *et al.* [3] used ADI value, developed on the basis of antimicrobial resistance of human intestinal microflora using the WHO guidelines. Different estimates of ADI values for other pharmaceutical compounds such as cyclophosphamide, acetylsalicylic acid are also shown in Table 3. These observations illustrate the importance of proper selection of endpoint for a given receptor.

At present, since most of the previous QPhRA studies have reported no appreciable human health risks associated with pharmaceuticals in water, the diversified choices of health endpoints do not essentially make a significant difference in risk characterization. However, if new circumstances emerge, different choices of endpoint might lead to different results of risk characterization, and even different decisions by risk management groups. From this point of view, it might be necessary to invest efforts to standardize or give authoritative reference on the general choice of endpoints of pharmaceuticals in water regarding QPhRA studies, or even provide reference values of ADIs.

During determination of endpoints, proper consideration of receptor’s susceptibility to the particular pharmaceutical is also required. For pharmaceuticals, while the therapeutic effect is considered beneficial for patient population, no benefit is presumed to be received by the individuals incidentally exposed to pharmaceuticals via ingesting drinking water or consuming fish, and hence it is often treated as an adverse effect in many QPhRA studies. For some pharmaceuticals that are developed for just one gender or age class, the therapeutic dose for the target population may not be the appropriate point-of-departure (POD) for calculating values of ADI for the non-targeted population, and consequently they may need individual evaluation [3,8]. Although the attention is generally given to the most sensitive adverse effect and sometimes, the lowest therapeutic dose has been used as the most “sensitive” POD for estimating values of ADI [3,8,10,12], this approach does not represent the effect of a pharmaceutical on a specific subpopulation type. For example, Kumar and Xagoraraki [10] used two types of ADI values (*i.e.*, toxicity- and therapeutic-based ADI values) for both adults and children subpopulations for estimating risks due to exposures of carbamazepine, meprobamate, and phenytoin from stream water or finished drinking water. For proper characterization of risk estimates, this approach appears to be preferable as it provides a better understanding about characterization of risk estimates.

In addition, special considerations are also required for some classes of pharmaceuticals, such as antibiotics (*i.e.*, with non-human target effects; for example: trimethoprim, tetracycline, oxytetracycline, doxycycline), chemicals with therapeutic dose above a toxic dose (*i.e.*, cytotoxic effect; for example: cyclophosphamide), chemicals which have high allergenic responses (for example: benzyl penicillin, phenoxymethyl-penicillin), or chemicals with high bioaccumulation potentials (for example: 17α-estradiol) ([3,7,12,17], Table 3). For example, cancer risk exists at any concentration levels of cyclophosphamide thus the therapeutic-based benchmark cannot be used for this pharmaceutical compound [17,33]. Antibiotics present a cause of concern due to their reported occurrence in environmental media and due to their potential for inducing antibiotic resistance. Although sufficient margin-of-safety has been observed during exposures of these pharmaceuticals from the aquatic environment [8,17], proper consideration and risk estimation are required for the case of occurrence of very high levels of antibiotics in wastewater effluents as reported recently by the Larsson *et al.* [14] and Phillips *et al.* [28] studies (concentration: >1,000 μg/L). Proper considerations are required during estimation of POD for pharmaceuticals with regards to their pharmacological or allergenic effects once their therapeutic effects subside. Overall, values of PODs should be estimated based on interaction of pharmaceuticals with endpoint-under consideration for a given subpopulation. Further, PODs should not be used interchangeably for different subpopulations, unless assumptions and conditions are documented adequately.

#### 2.3.3. Sensitive subpopulation

Proper considerations of gender or age class are also required during estimation of representative ADI for QPhRA for different sensitive subpopulations (*i.e.*, pregnant women, elderly, and children). For some pharmaceuticals that are developed for just one gender or age class, the therapeutic dose for the target population may not be the appropriate point-of-departure (POD) for calculating estimates of ADI for non-targeted population. Although an uncertainty factor of 10 is usually used to account for the variability among humans, its strength in protecting the special subpopulation remains difficult to verify for different pharmaceuticals, found in drinking water sources, thus these subpopulations need individual evaluations [3,6,9,10,11].

#### 2.3.4. Mixture effects

As occurrence of multiple pharmaceuticals in water at low concentrations have been reported [1,4,13,23,33], consideration of their interactions in QPhRA becomes important as it constitutes an important uncertainty [3,8,11]. Due to lack of understanding about (1) actual composition of pharmaceutical mixtures and (2) toxicity of pharmaceuticals at low concentration levels in mixture of other pharmaceuticals, it becomes difficult to predict bodily responses to mixture of pharmaceuticals. A review of QPhRA studies presented in Table 1 indicates that so far, most of the QPhRA studies have considered risk assessment due to individual pharmaceuticals and none of them have considered effect of mixtures of different pharmaceuticals. Recently Kumar and Xagoraraki [10] used information about carbamazepine, meprobamate, and phenytoin provided by the RxList to understand their interaction with each other using a pair of two active pharmaceutical ingredients (APIs) (Table 1) and qualitatively discussed the potential effect of simultaneous presence of different APIs. Although this approach appears to serve the purpose of understanding the interactive effect of APIs, it does not help in getting quantitative risk estimates.

To circumvent the issue of QPhRA of mixture of pharmaceuticals in water, studies have generally discussed different assumptions following the U.S.EPA [33] guideline for health risk assessment of chemical mixtures. Further, due to the present use of consideration of different UFs for estimation of HBLs and its subjectivity, the current QPhRA methodology overestimates risk estimates and is expected to compensate the effect of simplified assumption of consideration of no mixture effect on risk estimates.

Due to the potential additive, antagonistic, or synergistic nature of pharmaceuticals, any comprehensive risk assessment method addressing the issue of mixture effects is expected to be complicated [11,18]. Generally, the additive effect due to different pharmaceuticals is expected if pharmaceuticals act through the same mechanism [34]. It is worth noting here that Cleuvers [33] reported that even at concentrations at which the single substance showed no or only very slight effects, toxicity of the mixture was considerable [33]. Further these effects could be concentration-dependent as Pomati *et al.* [35] observed during their toxicity study using 13 drugs. A summary of these toxicity studies using mixtures of chemicals is presented in Table 4. Although most of these studies have assessed toxicity using aquatic indicator species or non-specific tests [21,33,34], findings of these studies provide perspectives about affects due to presence of different pharmaceuticals at different levels. For example, findings of Cleuvers [33] or Pomati *et al.* [35] are useful in conducting ecological risk assessment for aquatic species due to mixture of these pharmaceuticals within the range of concentration levels studied. Recently, Watts *et al.* [19] considered mixture toxicity quantitatively in QPhRA and estimated exposure ratio (*i.e.*, ratio of minimum therapeutic dose (MTD) to environmental dose) for total of 19 non-steroidal-anti-inflammatory-drugs (NSAIDs) by combining their exposure dose values and using lowest value of MTD (*i.e.*,7.5 mg for meloxicam), illustrating the approach for addressing mixture effects in QPhRA quantitatively.

In general, more toxicological work is required to study interactive effects of different pharmaceuticals present in water on different end points. To use the observed mixture effects data (Table 4) for conducting QPhRA for humans, we propose to use a composite uncertainty factor representing effect of mixture of pharmaceuticals on endpoint of a particular pharmaceutical, *i.e.*, “mixture effects-related uncertainty factor” (*mixUF_composite_*, hereafter). The *mixUF_composite_* parameter consists of all different types of uncertainty factors for a given mixture of pharmaceuticals (*i.e.*, *mixUF_i_*), similar to five uncertainty factors used for a single pharmaceutical (Section 2.3.1) and an additional uncertainty factor representing consideration for concentration levels of different pharmaceuticals in mixture (*i.e.*, *mixUF*_6_). Using Equation (1), values of *mixUF_composite_* for a given pharmaceutical in a mixture of pharmaceuticals could be calculated and used further to calculate POD using relationships previously used for a single pharmaceutical compound [3].

(1) $${mixUF}_{composite}=\sum_{k=1}^{6}{mixUF}_{k}$$

Currently, due to lack of detailed knowledge about different *mixUF_i_* (i = 1 to 6), further research efforts should be focused on getting values for these uncertainty factors for handling issues of mixture effects of pharmaceuticals in QPhRA. Further exploration of Quantitative Structure-Activity Relationship (QSAR) modeling techniques and other toxicogenomics and probabilistic approaches [26] could possibly help in understanding and determining values of uncertainty factors for mixture of pharmaceuticals.

## 3. Conclusions

This study reviewed different QPhRA studies to identify existing issues and proposed possible suggestions to address these issues, as summarized in Table 5. In general, for low concentrations of APIs, none of the QPhRA studies has identified any human health risks via exposure to drinking water, but uncertainties related to the QPhRA still exist and warrant consideration. The existing findings do not rule out the possibility of any human health. As the present risk values are estimated based on very limited knowledge about chronic effects and mixture effects of pharmaceuticals, this study proposes a development of a new “mixture effects-related uncertainty factor” for mixture of pharmaceuticals, similar to an uncertainty factor used for a single chemical within the QPhRA framework. In addition to all five traditionally used uncertainty factors, this factor is also proposed to include concentration effects due to presence of different concentration levels of pharmaceuticals in a mixture. However, further work is required to determine these factors and incorporate them within the QPhRA framework.

## Figures and Tables

**Table 1:** Summary of previous quantitative pharmaceutical risk assessment studies for ingestion of pharmaceuticals in water.

Reference (Country)	Hazard Identification	Exposure Assessment	Dose-response Relationship	Risk Characterization and Conclusions
Christensen [7] (Denmark) (Academia)	17α-ethinylestradiol (EE2), phenoxymethylpenicillin (Pen V), Cyclophosphamide (CP)	Intake of drinking water, leaf crops, root crops, fishes, dairy products, and inhalation of air; Only one subpopulation type (adults: 70 kg body weight)	For EE2: Higher intake from fish than other medium; For PenV and CP: Higher intakes from drinking water than other medium For PenV-at least 10 international units of penicillin (5.9 μg penicillin) is required to trigger a mode of action. For CP-toxic endpoint is genotoxicity (*i.e.*, a genotoxic carcinogen) No consideration for mixture effect	Negligible human risks; For the case of PenV-considerations are required for sensitive population which may react with few molecules; More risk assessment studies for other veterinary drugs
Schulman *et al.* [11] (Europe) (Academia)	Acetylsalicylic acid, clofibrate, cyclophosphamide, indomethacin	Ingestion of water and consumption of fish Water ingestion rate: 2 L/d (adults); Fish ingestion rate: 0.0178 kg/d general population and sports anglers, 0.039 kg/d subsistence fishers; Body weights: 70 kg (adults) and 30 kg(children)		No health risks
Webb *et al.* [12] (Europe) (Academia and Industry)	Acetyl salicylic acid, clofibric acid, ibuprofen, gemfibrozil, fenoprofen, ketoprofen, diclofenac, fenofibric acid, bezafibrate, indometacine, salicylic acid, atenolol, sotalol, salbutamol, terbutalin, fenoterol, nadolol, metropolol, celiprolol, carazolol, clenbuterol, phenazone, ifosfamide, cyclophosphamide, carbamazepine, pentoxifylline, clofibrate, phenazone, dimethlaminophenazon, ifosfamide, cyclophosphamide, carbamazepine, pentoxifylline, diazepam, fenofibrate, etofibrate, clarithromycin, dehydrato-erythromycin, roxithromycin, sulfamethazine, sulfamethoxazole, trimethoprim, chloramphenicol, chloroteracycline, doxycycline, tetracycline, cloxacillin, dicloxacillin, methicillin, nafcillin, oxacillin, benzylpenicillin, phenoxymethylpenicillin, iopamidol, iopromide, ioxithalamic acid, iothalamic acid, diatrizoate, 17α-Ethinylestradiol	Ingestion of water Water ingestion rate: 2 L/d (adults);	Comparisons of daily intake values (or dose equivalent for exposures for 70 years) with minimum daily therapeutic dose; No consideration for mixture effect	Low possibility of health risks
Schwab *et al.* [3] (U.S.A.) (Industry)	Acetaminophen, ablution, cimetidine, ciprofloxacin, codeine, dehydronifedipine, digoxigenin, digoxin, diltiazem, doxycycline, enalaprilat, erithromycin-H2O, fluoxetine, gemfibrozil, ibuprofen, lincomycin, metformin, norfloxacin, oxytetracycline, paroxetine metabolite, ranitidine, sulfamethoxazole, sulfathiazole, tetracycline, trimethoprim, warfarin	Ingestion of surface water and consumption of fish Water ingestion rate: 2 L/d (adults) and 1 L/d (children); Fish ingestion rate: 0.0175 kg/d (adults) and 0.0065 kg/d (children); Both subpopulation types with body weights = 70 kg (adults) and 14 kg (children); also inclusion of elderly and infirm where pharmacologic effect is undesirable Non-volatile active pharmaceutical ingredients => inhalation exposure is neglected; Similarly, dermal exposure pathways is assumed to contribute smaller risk compared to incidental ingestion risks	Use of both measured and predicted environmental concentrations and comparison of these values with calculated predicted-no-effect concentration (PNEC) values (separately for consumption of drinking water, fish, and for simultaneous consumption of both drinking water and fish) No consideration for mixture effect	No appreciable risks to humans Due to smaller values of bioconcentration factors, fish consumption is unlikely to be major exposure pathway
Watts *et al.* [19] (U.K.) (Industry and Academia)	396 pharmaceuticals and 11 illicit drugs	Ingestion of surface water Water ingestion rate: 1 L/d (adults) (no considerations for sensitive subpopulation) Calculation of surface water pharmaceutical concentration using the European Medicines Agency (EMA) models	Comparison of minimum therapeutic dose (MTD) with estimated exposure dose Quantitative consideration for mixture effect	No human health risks (margin-of-safety observed >1,000 for most of the drugs except for some drugs where ratios were observed to be below 100, when combination of 19 NSADI were used with MTD value.
Bercu *et al.* [6] (U.S.A.) (Industry)	Atomoxetine, duloxetine, olanzapine (neuropharmaceutical compounds)	Ingestion of surface water and consumption of fish Water ingestion rate: 2 L/d (adults) and 1 L/d (children) Fish ingestion rate: 0.0175 kg/d (adults) and 0.013 kg/d (children) Body weights: 70 kg (adults) and 30 kg(children) Calculation of surface water pharmaceutical concentration using the USFDA (1998) and PhFATE surface water models	Determination of lowest relevant effect dose using preclinical and clinical studies and its use for calculation of ADI; Use of human study for estimation of point-of-departure (POD) if effect is same from both animal and human studies and use of animal study with uncertainty factors for estimation of POD. Use of default uncertainty factors in the case of insufficient data for extrapolation purposes and where applicable, use of clinical trial data (*i.e.*, chemical- specific-adjustment factors) accounting human variability Calculation of predicted no- effect concentration (PNEC) in water, assuming that ADI could be delivered through drinking water and by eating fish No consideration for mixture effect	No appreciable health risks
Snyder [18] (U.S.A.) (Government)	Atenolol, atorvastatin, carbamazepine, diazepam, diclofenac, enalapril, fluoxetin, gemfibrozil, meprobamate, naproxen, phenytoin, risperidone, simvastatin, sulfamethoxazole, triclosan, trimethoprim			No health risks
Johnson *et al.* [20] (UK) (Academia)	Cytotoxic chemotherapy drugs (5-fluorouracil, cyclophosphamide, epirubicin/doxorubicin)	Ingestion of water Water ingestion rate: 1.5 L/d (adults) Body weight: 70 kg (adults)	Prediction of concentration for 5-fluorouracil and comparison with 10 ng/L trigger cytotoxic concentration value for humans; and comparison with PNEC value for aquatic fauna No consideration for mixture effect	No appreciable health risks
Rowney *et al.* [17] (UK) (Academia)	Three cytotoxic drug groups: alkylating agents (oxaliplatin, temozolomide, cisplatin, carboplatin, cyclophosphamide), antimetabolites (gemcitabine, fludarabine, fluorouracil-a metabolite to the prodrug capecitabine), and anthracycline antibiotics (epirubicin, doxorubicin).	Calculation of predicted environmental concentration using information, such as drug use, excretion rate and removal in sewage treatment plant and dissipation (assumed to be negligible) and dilution considerations in stream water Ingestion of raw stream water (using predicted drug concentrations for mean and 90th percentile flow conditions); Water ingestion rate: 2 L/d (adults)	Comparison of PEC estimates with threshold-of-toxicological- concern (TTC) and no-significant-risk-levels (NSRLs). No consideration for mixture effects	No health risks
Cunningham *et al.* [8] (U.S.A.) (Industry)	44 active pharmaceutical ingredients marketed by GlaxoSmithKline	Ingestion of water and consumption of fish Water ingestion rate: 1.5 L/d (adults) and 1 L/d (children); Fish ingestion rate = 0.0065 kg/d Both subpopulation types with body weights = 70 kg (adults) and 14 kg (children)	Use of both measured and predicted environmental concentrations and their comparisons with PNEC levels; Use of threshold of toxicological concern No consideration for mixture effect	No appreciable health risks
Crider *et al.* [21] (U.S.A.) (Academia)	Penicillins, erythromycins, cephalosporins, sulfonamides, quinolones, tetracyclines, aminoglycosides, and nitrofurantoins	Exposure : Reported maternal use of these antibiotics (1 month before the pregnancy through the end of the first trimester); A retrospective case-control study	Calculated Odds ratio and measured association of antibiotic use and selected birth defects No consideration for mixture effect	Penicillins erythromycins, cephalosporins: No association with birth defects. Sulfonamides, nitrofurantoins: Association with several birth defects
Schriks *et al.* [22] (Netherlands) (Academia)	50 chemicals in surface, groundwater, and drinking water	Water ingestion rate: 2 L/d (adults) Adult subpopulation with 70 kg body weight	Compared environmental concentration with threshold of toxicological concern For chemicals in surface water and groundwater, a benchmark quotient of 0.2 is used as chemicals are removed in drinking water treatment plants also. No consideration for mixture effect	No appreciable health concern
Cunningham *et al.* [9] (U.S.A.) (Industry)	Carbamazepine and its metabolites (carbamazepine diol and carbamazepine N-glucuronide)	Ingestion of water and consumption of fish Water ingestion rate: 1.5 L/d (adults) and 1 L/d (children); Fish ingestion rate = 0.0065 kg/d Both subpopulation types with body weights = 70 kg (adults) and 14 kg (children) (consideration for sensitive subpopulation)	Use of both measured and predicted environmental concentrations and their comparisons with PNEC levels; Use of threshold of toxicological concern No consideration for mixture effect	No appreciable health risks
Kumar and Xagoraraki [10] (U.S.A.)/(Academia)	Carbamazepine, phenytoin, meprobamate in U.S. surface water and finished drinking water	Ingestion of water and consumption of fish during recreational activity and direct consumption of finished drinking water Water ingestion rate: 0.1 L/d for surface water and 2 L/d for finished drinking water; Fish ingestion rate = 0.020 kg/d Both subpopulation types with body weights = 70 kg (adults in 18–75 years age group) and children of 1–10 years age group with body weight (kg) = 8 + 2 × Age)	Use of sub-population specific toxic endpoints; Use of calculated therapeutic- and toxicity- based acceptable daily intake values Consideration for mixture effect qualitatively (No reported interactions of meprobamate with phenytoin and carbamazepine at therapeutic concentration levels; Reported interaction of carbamazepine with phenytoin (decrease in pharmaceutical concentration due to presence of other pharmaceutical compound)	No appreciable health concern

**Table 2:** Removals of emerging organic chemicals in drinking water treatment plans: (a) Conventional treatment: Combination of filtration (sand), clarification, GAC adsorption, and chlorination unit processes and (b) Advanced treatment: Combination of conventional treatment unit processes with ozonation, ultra-violet irradiation, membrane filtration unit processes) [1,4].

	Less than 90% removal	More than 90% removal
**Conventional treatment**		
**Name of chemicals**	4-nonylphenol; 7-Acetyl-1,1,3,4,4,6-Hexamethyl-1,2,3,4- tetrahydronaphthalene (AHTN); bisphenol A; codeine; dehydronifedipine; Diethoxyoctylphenol (OP_2_EO); methylbenzyldene camphor; sulfathiazole; tri(2-butoxyethyl) phosphate; triclosan; triethylcitrate; Tris (2-chloroethyl)phosphate (TCEP)	17β-estradiol; acetaminophen; atorvastatin; benzophenone-3; carbamazepine; carbaryl; clofibric acid; diazepam; diazinon; diclofenac; erythromycin; estrone; fluoxetine; gemfibrozil; HHCB; lincomycin; metolachlor; naproxen; progesterone; sulfamethoxazole; trimethoprim

**Advanced treatment**		

**Name of chemicals**	4-nonylphenol; triclosan; TCEP	Atenolol; atrazine; bezafibrate; carbamazepine; clofibric acid; cotinine; diclofenac; estrone; gemfibrozil; ibuprofen; linuron; meprobamate; metolachlor; N,N-diethyltoluamide (DEET); naproxen; phenytoin; progesterone; sulfamethoxazole

**Table 3:** Summary of previous studies using different acceptable daily intake (ADI) values for the same pharmaceutical compound (Information about endpoints considered during estimation of ADI values are presented in parentheses).

Pharmaceutical name	Webb *et al.* [12] (assumed body weight = 60 kg)	Schwab *et al.* [3]	Schulman *et al.* [11] (assumed body weight = 60 kg)	Christensen [7]
Acetylsalicylic acid	8.3 μg/kg/d (30 mg/day therapeutic dose as anticoagulation therapy)	Not applicable	16.67 μg/kg/d	Not applicable
Cyclophosphamide	16.67 μg/kg/d (1 mg/d based on immunobullous skin disorders)	Not applicable	0.017 μg/kg/d (1 μg/d based on no-significant-risk-level)	0.01 μg/d (for rat)
Doxycycline	3 μg/kg/d (100 mg/day therapeutic dose based on bacterial infection)	30 μg/kg/d (value established from WHO representing antimicrobial sensitivity of human intestinal microflora)	Not applicable	Not applicable
Tetracycline	3 μg/kg/d (1,000 mg/day therapeutic dose based on bacterial infection)	30 μg/kg/d (value established from WHO representing antimicrobial sensitivity of human intestinal microflora)	Not applicable	Not applicable
Oxytetracycline	3 μg/kg/d (1,000 mg/day therapeutic dose based on bacterial infection)	30 μg/kg/d (value established from WHO representing antimicrobial sensitivity of human intestinal microflora)	Not applicable	Not applicable
17α-ethinylestradiol	0.167 μg/kg/d (0.010 mg/d therapeutic dose based on menopausal symptoms)	Not applicable	Not applicable	6 μg/d (prepubescent boys)
Phenoxymethyl-penicillin	16666 μg/kg/d (1,000 mg/day therapeutic dose based on bacterial infection)	Not applicable	Not applicable	5.9 μg/d
Clofibrate	8333 μg/kg/d (500 mg/d therapeutic dose based on hyperlipoproteinaemia)	Not applicable	278 μg/kg/d	Not applicable

**Table 4:** Literature-reported mixture effects of pharmaceuticals.

Reference	Component chemicals of mixture	Testing approach	Observed mixture effects
Silva *et al.* [34]	Eight chemicals of environmental relevance: 2′,3′,4′,5′-tetrachloro-4-biphenylol, 2′,5′-dichloro-4biphenylol, 4′-chloro-4-biphenylol, genistein, 2,4-dihydroxybenzophenone, benzyl-4-hydroxyparabene, bisphenol A, resorcinol monobenzoate	Recombinant yeast estrogen screen (YES)	There were substantial mixture effects even though each chemical was present at levels well below its NOEC and EC01.
Cleuvers [33]	Diclofenac, ibuprofen, naproxen, acetylsalicylic acid	Acute *Daphnia* and algal tests	Toxicity of the mixture was considerable, even at concentrations at which the single substances showed no or only very slight effects.
Pomati *et al.* [35]	A mixture of 13 different drugs at environmentally relevant concentrations: atenolol, bezafibrate, carbamazepine, ciprofloxacin, cyclophosphamide, furosemide, hydrochlorothiazide, furosemide, hydrochlorothiazide, ibuprofen, lincomycin, ofloxacin, ranitidine, salbutamol, sulfamethoxazole	*in vitro* cytotoxicity in *Escherichia coli*, human embryonic HEK293, and estrogen-responsive OVCAR3 tumor cells	(1) Drugs could interact and behave as chemosensitizers, with joint effects representing a statistically significant element of mixture toxicity. (2) Effects and interactions were concentration dependent.

**Table 5:** Summary of identified issues related to QPhRA and possible suggestions.

Issue	Issue description	Research needs/Suggestions
Measured *versus* predicted pharmaceutical concentration	Very few predictive models for pharmaceutical concentrations have been validated [8,9,24,25]; It is difficult to model low-detected pharmaceuticals.	Validate models using measured concentrations ; Conduct uncertainty analysis of risk estimates to address issue of low detection.
Pharmaceuticals-of-concern	The list of both parent compounds and metabolites is consistently increasing [12,25,28] and it becomes difficult to conduct QPhRA for all detected compounds.	Update pharmaceuticals list and integrate prioritization approach with the QPhRA framework [36].
Pharmaceuticals needing special attention	Therapeutic dose-based POD estimates might not represent effects of anti-neoplastics, antibiotics, bioaccumulative, allergens, and metabolites on different subpopulations [7,8,9,3,12].	Consider final effects of these pharmaceuticals on different receptors during estimation of POD and conduct group-specific QPhRA for these pharmaceuticals.
Source water versus finished drinking water	Use of source water pharmaceutical concentration for risk estimation as a conservative approach for exposures to pharmaceutical from finished drinking water [6,8,25,12].	Conduct water source-specific QPhRA; Use source water pharmaceutical concentration as finished drinking water pharmaceutical if data on pharmaceutical concentration in finished drinking water is missing.
Exposure route	Assumed dominance of oral ingestion route compared to other indirect ingestion- or inhalation-related exposure routes [2,3,12].	Conduct pharmaceutical class-specific comprehensive QPhRA studies using all exposure routes for a given receptor.
Values uncertainty factors (UFs)	Uncertainty exists due to different choices of values of UFs [3,6,12].	Use chemical-specific adjustment factors (CSAFs) [6,26]; Use default UF values only if CSAFs are not available. Conduct long-term toxicity studies or combination of experiment-simulation based studies to predict long-term toxicity using short-term toxicity data to address the issue of uncertainty related to short-term/long-term extrapolation.
Sensitive subpopulation	For some pharmaceuticals that are developed for just one gender or age class, the therapeutic dose for the target population may not be the appropriate point-of-departure (POD) for calculating estimates of ADI for non-targeted population (*i.e.*, pregnant women, elderly, children)	Use subpopulation-specific POD values [3,6,8,9,11,12]; Use uncertainty factor equal to 10 only in the absence of subpopulation-related endpoints information.
Mixture effects	Co-occurrence of different pharmaceuticals in water may affect risk estimates.	Discuss all assumptions involved during QPhRA for mixture of pharmaceuticals [33]. Conduct more toxicity studies to develop mixture effects-related uncertainty factors.
